# A Case Report of a Rare Debilitating Complication of Diabetes: Neuropathic Cachexia

**DOI:** 10.7759/cureus.28845

**Published:** 2022-09-06

**Authors:** Merilyn Jerry, Nikhila Chelikam, Pheba Elias Mathew, Henok D Regassa, Praisy Jerin Chacko, Srikanth Puli, Badal Thakkar, Ann Baby, Ritvika Panghal

**Affiliations:** 1 General Medicine, West China Hospital, Sichuan University, Chengdu, CHN; 2 Clinical Research, Icahn School of Medicine at Mount Sinai, New York City, USA; 3 General Medicine, China Three Gorges University, Yichang, CHN; 4 General Practice, St. Paul’s Hospital Millennium Medical College, Addis Ababa, ETH; 5 Internal Medicine, West China Hospital, Sichuan University, Chengdu, CHN; 6 Internal Medicine, Dartmouth Hitchcock Medical Center, Lebanon, USA; 7 Internal Medicine, Sinai Hospital of Baltimore, Baltimore, USA; 8 Endocrinology, Highland Medical, PC, Nyack, USA; 9 Internal Medicine, Indiana University Health Ball Memorial Hospital, Muncie, USA

**Keywords:** orthostatic hypotension, fiber loss, burning sensation on soles, unintended weight loss, diabetic peripheral neuropathy (dpn), diabetic neuropathic pain, diabetes, cachexia

## Abstract

A 56-year-old male with a history of type 2 diabetes mellitus and hypertension presented with complaints of intractable burning paresthesia of bilateral extremities, hyperesthesia, and unintentional weight loss. Other symptoms included anorexia, orthostatic hypotension, bowel and bladder dysfunction, and painful burning sensation on the soles of the feet. Emotional lability and a melancholy mood were noted. After laboratory tests including CSF analysis, biopsies, and three months of treatment that did not bring relief, the patient was diagnosed with diabetic neuropathic cachexia (DNC). While his diabetes remained well-controlled, the patient was unable to improve his nutritional status and his condition progressively worsened, and he later died from cardiac arrest. DNC is an important differential diagnosis to consider in patients with neuropathy and weight loss. Early detection of DNC in conjunction with weight loss investigation may reduce pain and speed recovery with a good prognosis.

## Introduction

The association between diabetes and peripheral neuropathy has been known for over a century. Decades ago, due to the ambiguity of the origin and progressive nature of the painful neuropathy, it was identified as one of the most troublesome complications of diabetes [1]. Later, the spectrum of diabetic neuropathy was subdivided based on focal or diffuse distribution [2]. Distal symmetric sensorimotor polyneuropathy is the most common form of presentation, affecting almost half of all patients diagnosed with diabetes mellitus. The less common presentations of diabetic neuropathies include acute mononeuropathies, diabetic amyotrophy, autonomic neuropathies, and pressure neuropathies. Diabetic neuropathic cachexia (DNC), however, is a rare form of diabetic neuropathic presentation [2,3]. The term was first proposed by Ellenberg, because of the cachectic look in the affected patients [4]. DNC can be referred to as a specific neuroendocrine disorder in a patient with diabetes mellitus. It is characterized by extreme weight loss, acute or subacute onset of severe neuropathic pain over the limbs and trunk without associated weakness, and with or without sleep and mood disorders in a patient with diabetes mellitus [4]. In this report, we describe a case of DNC, which is one of the rarest presentations of diabetic neuropathy. Clinicians need to be aware of the presence of such debilitating complications of diabetes since it can be reversed when diagnosed and managed at an earlier stage.

## Case presentation

A 56-year-old male with a longstanding history of type 2 diabetes mellitus and systemic hypertension presented with complaints of intractable burning paresthesia of bilateral extremities with allodynia followed by unintentional weight loss of about 37 lbs over a period of two months. Other associated symptoms included anorexia, orthostatic hypotension, and bowel and bladder dysfunction. Hyperesthesia to touch was so marked that the mere pressure of clothes or a blanket brought on the pain. Though there was no motor weakness initially, as time progressed the patient developed generalized muscle wasting and loss of muscular strength. The gait was impaired by a painful burning sensation on the soles of the feet. Although mentally sound, there was significant emotional lability and a melancholy mood. An extensive evaluation was done to find the etiology of the significant weight loss and bilateral neuropathy.

Laboratory results included hemoglobin (Hb) 12.6 g/dl, WBC 10,600 per microliter, platelet count 295,000 per microliter, erythrocyte sedimentation rate (ESR) 42 mm, fasting glucose 103 mg/dl, glycated hemoglobin (HbA1c) 5.5%, vitamin B12 >2000, and normal folic acid and vitamin D levels. Urine analysis was noted to be normal, with a negative Bence-Jones protein urine test. Serum electrophoresis and serum electrolytes were within normal limits. Liver, kidney, and thyroid functions were also noted to be normal. Bone marrow aspiration cytology was performed in order to rule out any evidence of bone marrow malignancy as a likely etiology of his profound weight loss. CSF study showed cellular marrow with mild plasmacytosis (5-8%) and no abnormal cells were noted. CSF glucose was 77 mg/dl and the protein was 81 mg/dl. Immunofixation electrophoresis showed a faint IgA lambda positive band. Hematology consultation was sought for the presence of light chains in immunofixation, and a possible diagnosis of monoclonal gammopathy of undetermined significance (MGUS) was suggested. A positron emission tomography (PET) scan was done, which ruled out any bone lesion or organomegaly. An extensive workup was also done in view of the elevated ESR including Sjögren syndrome type A antigen (SS-A), Sjögren syndrome type B antigen (SS- B), antinuclear antibodies (ANA), rheumatoid factor (RF), C-reactive protein (CRP), Anti double-stranded (ds)DNA, Anti-myeloperoxidase (MPO), angiotensin-converting enzyme (ACE) levels, and complement levels, all of which came back negative. A minor salivary gland biopsy was also performed, which was negative for any vasculitis or amyloidosis. Viral makers for herpes simplex virus (HSV), HIV, hepatitis C virus (HCV), and hepatitis B virus (HBV) were all negative. Electromyography was performed, which showed normal nerve conduction studies. Skeletal muscle fiber biopsy showed mild fiber atrophy. Right superficial peroneal nerve biopsy with mild to moderate mild fiber loss. 

The patient was treated with intravenous cyclophosphamide 700 mg once a month for three months and a short course of low-dose steroids for the MGUS. A post-treatment repeat CSF study showed normal levels of plasma cells. Medications for neuropathic pain were also optimized. He was initially started on pregabalin 150 mg twice daily, duloxetine 20 mg twice daily, and amitriptyline 25 mg once daily, the dosage of which was further increased on follow-up as there was no therapeutic benefit achieved. Other medications included lorazepam 1 mg at bedtime, amlodipine 10 mg once daily, rosuvastatin 5 mg, glimepiride 2 mg, sitagliptin 50 mg, olmesartan 40 mg, and a daily dose of metformin SR 500 mg. The medications for neuropathic pain were later discontinued after three months as there was no relief of symptoms.

The diagnosis of DNC was made based on the clinical presentation of intractable symmetrical lower extremity pain, unexplained unintentional weight loss, anorexia, mood and sleep disturbances, and autonomic symptoms. Other differentials that were ruled out included diabetic amyotrophy, which is a painful asymmetric motor neuropathy but without associated mood disturbances or weight loss, and diabetic bulimia, which is associated with significant weight loss but this is intentional and seen in type 1 diabetes. 

The patient's condition progressively worsened with lower limb muscle wasting and the inability to walk due to severe pain. His diabetes remained well-controlled but he was unable to improve his nutritional status and there was no improvement reported in any of his symptoms. The patient had a cardiac arrest in his sleep and finally succumbed to his illness.

## Discussion

In 1974, Ellenberg first reported DNC in a group of six males in their sixth decade of life with characteristic features of bilateral symmetrical peripheral neuropathy, severe emotional disturbances, anorexia, impotence, mild diabetes, and the absence of other specific diabetic complications. The patients were noted to have spontaneous recovery in about one year [4]. So far over the years, less than 50 cases have been reported, and DNC has been found to be typically seen in middle-aged males, who are recently diagnosed with type 2 diabetes mellitus [5]. It is also known to occur irrespective of the duration of their diabetes, or type of diabetes (also seen in type 1 diabetes patients), in females, and in pediatric patients [6]. While impaired glycemic control and hyperlipidemia have been established as important in the pathogenesis of diabetic neuropathy [2], the precise pathogenetic mechanisms leading to the development of DNC are yet to be understood [7].

Majority of DNC patients present with acute to subacute bilateral symmetrical painful sensory-motor peripheral neuropathy, which spreads from the lower and upper extremities to the chest and abdomen. DNC patients can also present with autonomic neuropathic features like constipation, diarrhea, gastroparesis, and impotence [4]. Autonomic disturbances including diarrhea and orthostatic hypotension were found in our patient.

DNC was often considered synonymous with diabetic amyotrophy in regards to the motor manifestations like generalized wasting and decreased muscle strength. While the classic diabetic amyotrophy is characterized by proximal neuropathy, DNC has severe distal neuropathy, which is more pronounced in the proximal lower extremities than in the upper extremities without weakness and rapid weight loss [3,4]. The presence of proximal or truncal dysesthesia could be another clue suggesting the diagnosis of DNC [2]. While some cases are observed to have normal muscle strength, some have muscle atrophy and weakness [1]. Occasionally, sudden improvement in glycemic control, usually due to starting insulin treatment, could precipitate acute painful neuropathy [3].

Pain is characteristically burning in nature with predominant lower limb involvement and allodynia [3]. Patients with DNC may have severe contact hypersensitivity that is triggered by clothing or bedding, as well as stabbing or shooting pains in the legs. The pain is usually worse at night or when one is relaxing [1]. Our patient complained of intense unbearable pain, which was of sudden onset, in contrast to the gradual onset seen in peripheral neuropathy associated with diabetes.

The other characteristic feature of DNC is rapid, unintentional weight loss, the mechanism of which is so far unclear. Some reports attribute this weight loss to hyperglycemia leading to glycosuria or massive pain depression leading to anorexia [5]. The overall weight loss could reach up to 60% of total pre-neuropathic-onset body weight [4].

Other symptoms to be kept in mind are depression [1,2,4,8], anxiety [1,4,8], mood swings, and sleep disturbances along with anorexia [1,4]. While a majority of patients with DNC experience a single episode and recover fully, some recurrent cases also have been reported [9]. A comprehensive list of differential diagnoses including malabsorption syndromes, malignancies, connective tissue diseases (CTD), neuropathic carcinomatosis, toxic or alcoholic neuropathy, porphyria, and chronic relapsing Guillain-Barré syndrome must be ruled out before confirming the diagnosis of DNC [5,7].

Therapy of painful neuropathy is challenging. It must include both a selection of an appropriate agent for both mood stabilization and neuropathic pain control as well as psychiatric consultation for emotional support [8]. Medications like tramadol, dextromethorphan, topical capsaicin, clonidine, tricyclic antidepressants, and gabapentin are often effective. Opioids are rarely beneficial and may carry a high risk of addiction [3]. DNC is a partially reversible disorder [2]; early and optimal nutritional support, good glycemic control, and pain management can help in the improvement of symptoms and avoid further deterioration of DNC [3,7].

## Conclusions

Unfortunately, our patient did not show any improvement with several pain management medications in addition to not being able to improve his weight and nutritional status. Hence, it is important to reiterate the need for early nutritional support and weight gain in these cases for higher recovery rates. DNC is characterized mainly by rapid weight loss and severe painful neuropathic symptoms, so clinicians must be vigilant regarding excluding other possible diagnoses. It is also important to include a multidisciplinary team for quick diagnosis and management of DNC for a better prognosis.
